# Biomimetic open porous structured core-shell microtissue with enhanced mechanical properties for bottom-up bone tissue engineering

**DOI:** 10.7150/thno.34464

**Published:** 2019-07-09

**Authors:** Chao Luo, Huimin Fang, Muran Zhou, Jialun Li, Xinyue Zhang, Shaokai Liu, Chuchao Zhou, Jinfei Hou, Huan He, Jiaming Sun, Zhenxing Wang

**Affiliations:** Department of Plastic Surgery, Union Hospital, Tongji Medical College, Huazhong University of Science and Technology, Wuhan 430022, China.

**Keywords:** Core-shell, microtissue, bottom-up, demineralized bone matrix, biomimetic

## Abstract

**Background**: Microtissues constructed with hydrogels promote cell expansion and specific differentiation by mimicking the microarchitecture of native tissues. However, the suboptimal mechanical property and osteogenic activity of microtissues fabricated by natural polymers need further improvement for bone reconstruction application. Core-shell designed structures are composed of an inner core part and an outer part shell, combining the characteristics of different materials, which improve the mechanical property of microtissues.

**Methods**: A micro-stencil array chip was used to fabricate an open porous core-shell micro-scaffold consisting of gelatin as shell and demineralized bone matrix particles modified with bone morphogenetic protein-2 (BMP-2) as core. Single gelatin micro-scaffold was fabricated as a control. Rat bone marrow mesenchymal stem cells (BMSCs) were seeded on the micro-scaffolds, after which they were dynamic cultured and osteo-induced in mini-capsule bioreactors to fabricate microtissues. The physical characteristics, biocompatibility, osteo-inducing and controlled release ability of the core-shell microtissue were evaluated in vitro respectively. Then microtissues were tested in vivo via ectopic implantation and orthotopic bone implantation in rat model.

**Results**: The Young's modulus of core-shell micro-scaffold was nearly triple that of gelatin micro-scaffold, which means the core-shell micro-scaffolds have better mechanical property. BMSCs rapidly proliferated and retained the highest viability on core-shell microtissues. The improved osteogenic potential of core-shell microtissues was evidenced by the increased calcification based on von kossa staining and osteo-relative gene expression. At 3months after transplantation, core-shell microtissue group formed the highest number of mineralized tissues in rat ectopic subcutaneous model, and displayed the largest amount of new bony tissue deposition in rat orthotopic cranial defect.

**Conclusion**: The novel core-shell microtissue construction strategy developed may become a promising cell delivery platform for bone regeneration.

## Introduction

Tissue engineered bone grafts (TEBGs) are very promising for the treatment of large bone defects and fractures due to the few complications and disease transmission 1. Traditional TEBGs are fabricated by seeding cells on prefabricated scaffolds that provide environment cues for tissue development 2. Although many scaffolds have been developed, the treatment efficacy of traditional TEBGs for large bone defects is limited due to the following two reasons: (1) The uneven distribution of cells throughout the scaffold, wherein cells in the center die because of insufficient nutrients and oxygen transport to the center region 3; (2) Once implanted in vivo, cell loss due to mechanical disturbances, ischemic and inflammatory factors is inevitable 4.

Cells play important roles in determining the efficiency of TEBGs as they improve bone tissue regeneration by releasing osteo-inductive factors or directly differentiated into osteogenic cells 5. Bone marrow mesenchymal stem cells (BMSCs) have excellent characteristics such as high in vitro expansion capacity, low immunogenicity and robust osteogenic capacity, and thus are commonly used in bone tissue engineering 6. Therefore, microtissues formed by mesenchymal stem cells (MSCs) have been developed and are considered to be an alternative for traditional TEBGs 7.

Microtissues are characterized as single cell aggregates or micro-fabricated cell laden hydrogels 8. As for the fabrication of TEBGs, microtissues have the following advantages: Firstly, several recent studies have shown that microtissues can facilitate cell proliferation and specific differentiation because they possess many microarchitectural features that are similar to native tissues 8. Secondly, microtissues can assemble into a TEBG using the bottom-up (BU) strategy, in which large scale tissues are fabricated by building modular tissues first and then assembled 8. Similar to natural tissues made up of repeating functional units (osteon in bone, etc.^9^), TEBGs fabricated by BU strategy are structurally biomimetic wherein microtissues act as construction units. Thirdly, cells can migrate and reside in the internal spaces of microtissues through the open porous structures on the surface, and this protects the cells from the harsh microenvironment during early implantation 10. Despite the advantages mentioned above, currently used microtissues are often made up of natural hydrogels such as gelatin 11, which is unsuitable for hard tissue repair due to the lack of adequate mechanical property and osteogenic activity. Thus, microtissues need to be improved for TEBGs.

Core-shell structure is an emerging technology of scaffolding materials used in tissue engineering. The classical design of core-shell structure is a central part core and an outer part shell, and is endowed with many characteristics suitable for tissue engineering application 12. Core-shell structured scaffolds can be generated by various methods, such as co-concentric nozzle extrusion 13, microfluidics 14 and chemical confinement reactions 15. When used as scaffolds for tissue engineering, the advantages of core-shell structure are as follows: Firstly, core-shell structure combines the characteristics of two different materials 16. For example, Perez et al. utilized collagen-alginate core-shell microfibers as a cell delivery system for bone tissue engineering, in which the collagen-based core acted as an osteogenesis-phase and the alginate-based shell was used as an angiogenesis-phase 17. Secondly, cells or biomolecules can be loaded on the core and delivered to the object's defective site. Similarly, Huiyong Zhu et al. used bone morphogenetic protein-2 (BMP-2) encapsulated polyethylene glycol (PEG) as the core of a poly(caprolactone) (PCL) nanofibrous shell, achieving a zero-order release for over 24 days 18. Thirdly, the shell provides a favorable microenvironment for cells growth and acts as a niche that protects them from external impactful factors 19. Yanan Du et al. found that the primed gelatin micro-niches can act as a shelter to protect cells during injection, with 10^5^ human adipose-derived mesenchymal stem cells (hMSCs) seeded micro-niches realized better ischemic limb salvage than treatment with 10^6^ free injected hMSCs 20. Therefore, theoretically, the mechanical strength of microtissues can be improved when a material with enhanced mechanical property and osteogenic activity is used as the core. However, so far, core-shell structure has never been applied into microtissue construction.

Recently, much attention has been paid to natural polymers due to their biomimetic properties 21. Natural polymers are made up of fundamental elements such as C, N, Ca, H, O, etc., which are the same as the component of native extracellular matrix (ECM) 22. Moreover, the hierarchical structures of natural materials are similar to those of native tissue, which provides a biomimetic microenvironment for cells 23. Gelatin is a natural protein with aminoacidic sequences such as Arg-Gly-Asp (RGD), thus has a good biocompatibility and biodegradability profile 24. Demineralized bone matrix (DBM), a material with excellent mechanical property, osteo-conductivity and osteo-inductivity, is suitable for hard tissue treatment 25.

**Figure 1:**
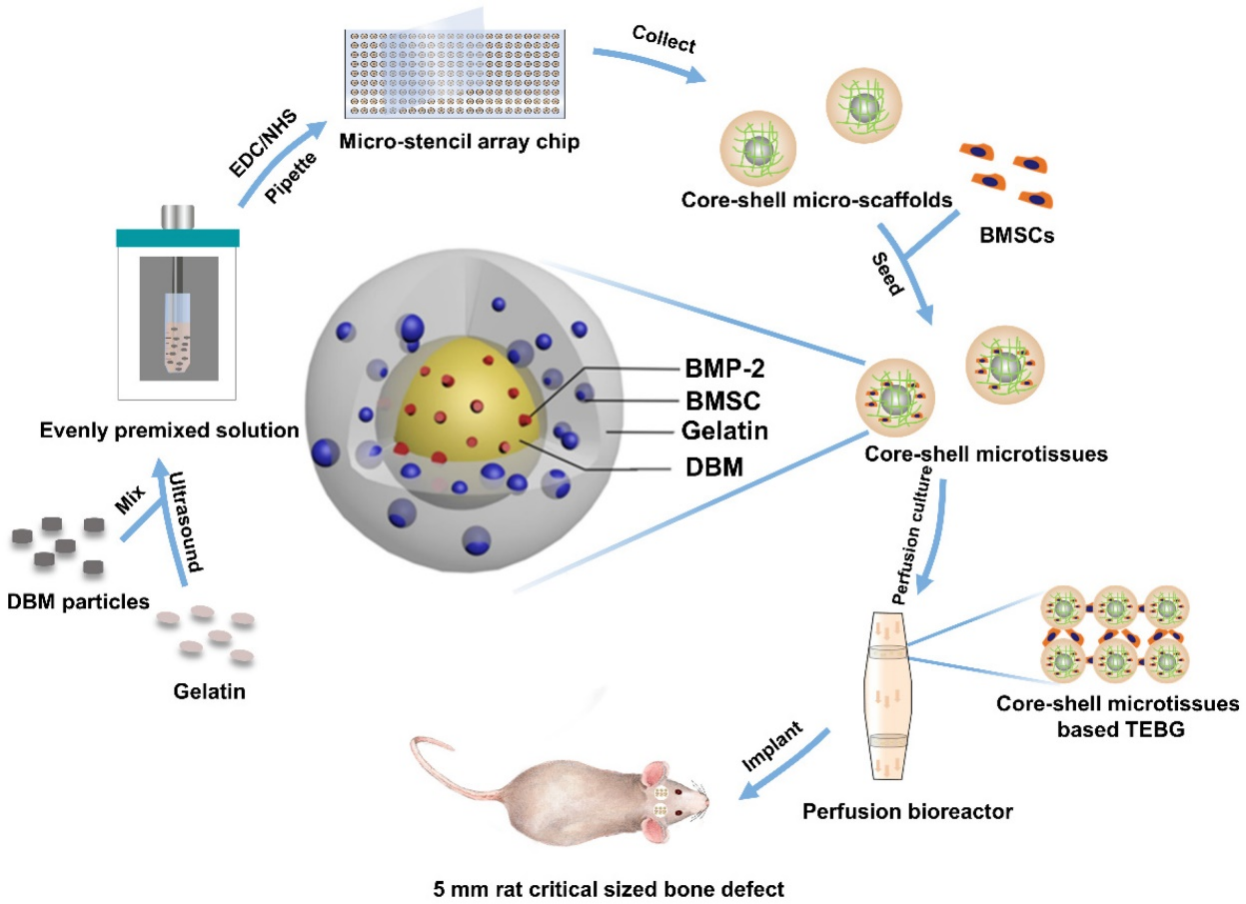
Diagram of experimental design. Briefly, 10 mg/mL DBM particles were added into 60 mg/mL gelatin solution. The mixture was then ultrasonically dispersed until DBM particles were uniformly distributed. EDC/NHS were added to crosslink the precursor solution, and the crosslinked precursor solution was then pipetted into micro-stencil array chip, after which the lyophilized core-shell micro-scaffolds in the chip were harvested using an Ejector Pin array. BMSCs were seeded onto core-shell micro-scaffolds to form core-shell microtissue which was composed of DBM core loaded with BMP-2 and a gelatin shell enclosing the core. After the dynamic expansion and osteo-differentiation period in the perfusion bioreactor, the core-shell microtissues assembled together to form TEBGs. The assembled TEBGs were then implanted into rat critical sized cranial defect site to evaluate its ability to repair the defect.

Against this background, we designed a biomimetic open porous structured core-shell microtissue with enhanced mechanical properties for BU bone tissue engineering as illustrated in **Figure 1** and **Video SI**. The DBM core loaded with BMP-2 not only provided a stiff base for cell adhesion, but also released BMP-2 to surrounding tissues to promote osteogenesis. Open porous gelatin shell provided a favorable microenvironment for BMSCs to grow, and acted as a protective niche for BMSCs during the early implantation period. Here, core-shell micro-scaffolds were first fabricated, characterized and benchmarked with gelatin micro-scaffolds, a commonly used micro-scaffold for tissue engineering. The viability of seeded BMSCs and the osteogenic activity of core-shell microtissues and gelatin microtissues were compared. Finally, whether the TEBGs fabricated by core-shell microtissues could improve the treatment of rat critical-sized cranial defect were assessed.

## Materials and Methods

### Animals

Male Sprague Dawley rats (4 weeks old, 150-200 g body weight) were purchased from the Department of Experimental Animals, Tongji Medical College, Huazhong University of Science & Technology (Wuhan, China). All animal experiments performed in this study were approved by the Department of Experimental Animals, Tongji Medical College, Huazhong University of Science & Technology (Wuhan, China). All animal procedures were consistent with state regulations and laws and in accordance with the Standing Committee on Ethics in China (State Scientific and Technological Commission of China). All animals were sacrificed after the experiment consistent with state regulations and laws and in accordance with the Standing Committee on Ethics in China (State Scientific and Technological Commission of China).

### Fabrication of core-shell micro-scaffolds and gelatin micro-scaffolds

Core-shell micro-scaffolds and gelatin micro-scaffolds were prepared by a micro-stencil array chip. The polymethyl methacrylate (PMMA) micro-stencil array chip was developed by laser prototyping technique as previously described 10. Micro-stencil array chips (261 circle wells with diameter of 1500 µm) were washed with deionized water. 60 mg/mL gelatin solution was then prepared by dissolving cold water fish gelatin (Sigma, St. Louis, USA) in deionized water at 50 °C. For core-shell micro-scaffolds, 10 mg/mL DBM particles (prepared as previously described 26) were added into the gelatin solution. The precursor solution (60 mg/mL of gelatin and 10 mg/mL of DBM particles) was maintained in ice-bath for 30 min and mixed using an ultrasonic processor (BRANSON, Danbury, USA). To crosslinking, 1% 1-(3-Dimethylaminopropyl)-3-ethylcarbodiimide hydrochloride (EDC, Sigma, St. Louis, USA) and 0.4% N-Hydroxysuccinimide;1-hydroxypyrrolidine-2,5-dione (NHS, Sigma, St. Louis, USA) were added into the precursor or gelatin solution and maintained at 4 °C for 1 h. After crosslinking, 200 µl precursor/gelatin solution was pipetted onto the micro-stencil array chip and scraped back and forth to ensure uniform distribution. The precursor/gelatin solution in the micro-stencil array chip was subjected to cryo-gelation for 16 h in -20 °C, followed by lyophilization process for 30 min (Boyikang, Beijing, China). Finally, core-shell micro-scaffolds/gelatin micro-scaffolds (diameter: 1500 µm) were harvested from the precursor/gelatin chip by a photopolymer Ejector Pin array. Both core-shell micro-scaffolds and gelatin micro-scaffolds were reserved in a vacuum with the shape of monolayer and lyophilized.

### Characterization of core-shell micro-scaffolds and gelatin micro-scaffolds

**General structure**: A phase contrast microscope (Ni-E, Nikon, Tokyo, Japan) was used to observe the general structure of gelatin micro-scaffolds, DBM particles and core-shell micro-scaffolds. To investigate the homogeneity of fabricated core-shell micro-scaffolds, the number of DBM particles in the core-shell micro-scaffolds was counted. The freeze-dried core-shell/gelatin micro-scaffolds and DBM particles were then coated with gold and observed under scanning electron microscope (SEM, S3400N, Hitachi, Tokyo, Japan) to evaluate their microstructures.

**Mechanical test**: To compare the Young's modulus of core-shell micro-scaffold and gelatin micro-scaffold, every 30 core-shell/gelatin micro-scaffolds were assembled as a cylinder by mixed fibrin gel (Laishi, Shanghai, China). The assembled micro-scaffolds (diameter: 5 mm, height: 2 mm) were compressed at a constant deformation rate of 16 µm/s with an all-electric dynamic test instrument (ElectroPuls E1000, INSTRON, Britain). Based on the strain limited to the first 30% and resulting stresses, the Young's modulus of the micro-scaffolds was calculated.

**Pore size and Porosity**: The pore size was evaluated from SEM images of seven different areas using Image-Pro Plus 6.0 software 20. To investigate the porosity of the core-shell micro-scaffolds and gelatin micro-scaffolds, a liquid displacement technique was used as described before 27. Briefly, equal number of freeze-dried core-shell/gelatin micro-scaffolds were soaked in deionized water with a known volume (V_1_) for 1 h. The total volume of water and micro-scaffolds were marked as V_2_. After removing the immersed samples, the volume of residual water was recorded as V_3_. The porosity was obtained by the following formula; (V_1_-V_3_)/(V_2_-V_3_) × 100%.

**Seeding efficiency and controlled release of BMP-2**: To investigate the seeding efficiency of micro-scaffolds, 30 core-shell/gelatin micro-scaffolds were placed into a polydimethylsiloxane (PDMS) chamber in 12-well cell culture plate. Prior to cell seeding, the micro-scaffolds were washed using sterilized PBS three times followed by an immersion in 75% ethanol for 1 h. This was followed by washing with PBS three times to remove ethanol. Equal number of BMSCs were seeded onto core-shell/gelatin micro-scaffolds (60 μL of 5×10^6^/mL for every 30 micro-scaffolds), which were then incubated at 37 °C for 2 h. Samples were subsequently washed with PBS twice, the number of residual BMSCs was calculated by subtracting those washed out by PBS 27.

To investigate the controlled release of BMP-2, same quantity of DBM particles/core-shell micro-scaffolds were placed into 10 mL of PBS and shaken. The PBS was replaced every 2 days with 10 mL of fresh PBS. After 26 days, PBS was collected and analyzed by a BMP-2 Quantikine ELISA kit (R&D Systems, UK).

### Isolation and culture of rat BMSCs

Newborn Sprague Dawley rats (5-7 days old) were sacrificed by cervical dislocation. The rats were then immersed in 75% alcohol for 10 min before the ends of the tibiae and femurs were cut and separated from surrounding muscles and soft tissues. The bone marrow was flushed out repeatedly with low glucose Dulbecco's Modified Eagle's Medium (DMEM, HyClone, Logan City, UT, USA) containing 10% FBS (Hyclone, Logan City, UT, USA), 1% penicillin and streptomycin (Thermo Fisher Scientific, Waltham, MA, USA). Cells were cultured on dishes at 37 °C under 5% CO_2_ conditions, and culture medium was changed every 3 days. When the attached cells reached 80%-90% confluence, they were digested with 0.25% trypsin/1 mM EDTA (Thermo Fisher Scientific) and passaged.

### Integrated fabrication (dynamic expansion and osteo-differentiation) of core-shell microtissues-based TEBGs

**Design of mini-capsule perfusion bioreactor**: The bioreactor was developed as schematically shown in **Figure 3A, Figure 3B**. The bioreactor consisted of three parts: a peristaltic pump (Huiyu, Beijing, China) as the driving part, a medium reservoir and a mini-capsule flask. As shown in **Video. SI**, the mini-capsule flask contains two discrete parts which are connected by the interlocking threads. The flask can hold 4 polylactic acid (PLA) honeycomb like chambers fabricated using 3D printing technology. The honeycomb like chamber was mainly composed of a seal cover and a base plate with pores (120 µm in diameter) to allow perfusion (**Figure 3B**). Each base plate comprises 5 duplicate perfusion chambers of 5 mm diameter and 2 mm height.

**Dynamic expansion:** The core-shell micro-scaffolds and gelatin micro-scaffolds were sterilized by soaking in 75% ethanol for 30 min, followed by immersion in 1% gelatin solution for 12 h. 30 sterilized core-shell/gelatin micro-scaffolds were then assembled in a perfusion chamber of the honeycomb like chamber. BMSCs at passage 3 were digested with trypsin and resuspended at a cell concentration of 5×10^6^ cells/mL. Every 30 core-shell/gelatin micro-scaffolds were seeded with 60 µl of cell suspension and incubated at 37 °C for 2 h. Honeycomb like chambers containing cells seeded core-shell/gelatin micro-scaffolds were transferred into the mini-capsule flask. Media cycling in the bioreactor was continuously pumped through the micro-scaffolds. The flow rate was set as 2 mL/min and the media were replaced every 3 days.

**Dynamic osteo-differentiation**: During the expansion period, the viability of seeded cells reached the top at day 7. Osteogenic differentiation medium (low-glucose DMEM supplemented with 10 mM β-glycerophosphate, 10-8 M dexamethasone, and 0.2 mM ascorbic acid (Sigma, St. Louis, USA) was added instead of culture medium after 7 days of expansion. The osteo-differentiation medium was changed every 3 days and the differentiation period was sustained for another 21 days. After the dynamic expansion and osteo-differentiation, BMSCs were proliferated and differentiated into osteoblasts on micro-scaffolds to form microtissues (**Table S1**).

### In vitro biocompatibility assessment

**FDA/PI assay:** Fluorescein diacetate (FDA)/ propidium iodide (PI) staining was performed to evaluate live and dead cells in microtissues at the corresponding time points (12 h, day 3, day 7 and day 10). Briefly, samples were collected from the perfusion chamber, washed three times by PBS. Then, 1mL of 2 μg/mL FDA (Sigma, St. Louis, USA) was added and incubated at 37 °C for 30 min to stain the cytoplasm of living cells green. After washing three times with PBS, 1mL of 100 μg/mL PI (Sigma, St. Louis, USA) was added and incubated at room temperature to stain the nuclei of dead cells red. Stained samples were observed under a confocal laser microscope (Leica Microsystems, Wetzlar, Germany).

**SEM analysis:** After seeding cells for the corresponding period (2 h, day 3, day 7, day 10), microtissues were removed from the 6-well plates. They were washed with PBS twice and fixed in 4% (vol/vol) glutaraldehyde for 24 h followed by a gentle washing three times and then dehydrated using serial concentrations of ethanol (50%, 75%, 80%, 90% and 100%). After air drying and sputtering with gold, the morphology of the seeded BMSCs and accumulation of ECM were observed by SEM (S3400N, Hitachi, Tokyo, Japan).

**MTT assay:** MTT assay was performed In accordance with the manufacturer's protocol to evaluate the activity of loaded BMSCs at different time points (12 h, day 3, day 7 and day 10) 28. Microtissues were mixed with 50 μL of 3-(4,5-dimethylthiazolyl-2)-2,5-diphenyltetrazolium bromide (MTT, 5 mg/mL in PBS, Sigma, St. Louis, USA) and 100 μL of growth medium, then incubated at 37 °C in 5% CO_2_ in darkness for 4 h. The microtissues were then washed and treated with 150 μL of dimethyl sulfoxide (DMSO, Sigma, St. Louis, USA) to extract formazan crystals. Finally, a DU 730 UV/Vis spectrophotometer (Beckman Coulter) was used to detect the 570 nm absorbance (optical density, OD).

**Quantification of dsDNA content**: Every 30 core-shell/gelatin microtissues were collected at the same time points as mentioned above. Then, BMSCs on microtissues were digested with 0.25% trypsin/1 mM EDTA (Thermo Fisher Scientific). After centrifugation, the dsDNA content of BMSCs on 30 core-shell/gelatin microtissues were measured by the following steps: DNA purification using TIANamp Gennomic DNA Kit (DP304, TIANGEN BIOTECH, China, Beijing) and DNA quantification using Quant-iT™ Picogreen™ dsDNA Assay Kit (P7589, Invitrogen, Carlsbad, Calif, USA).

### Von Kossa and calcium content

**Von Kossa**: Von Kossa assay was performed to determine the mineralized nodules on microtissues as described 29. After washing twice with PBS, samples were fixed in 4% paraformaldehyde for 1 h. This was followed by rinsing with double distilled water and incubating with 2% AgNO_3_ in darkness for 10 min, followed by exposure to UV light for 15 min. Finally, samples were observed under a camera equipped with a close-up filter.

**Calcium content**: Thirty microtissues was harvested at the corresponding time points (12 h, day 7, day 14, day 21) after changing the osteogenic medium. The samples were washed twice with PBS and immersed in 0.5 mL of 10% acetic acid to isolate calcium from the microtissues. The samples were then tested using the Calcium Assay Kit (Jiancheng, Nanjing, China). Finally, specimens were observed under a DU 730 UV/Vis spectrophotometer (Beckman Coulter) and the absorbance at 610 nm was measured (optical density, OD).

### Reverse transcription polymerase chain reaction

Relative expression of bone related genes was evaluated by qRT-PCR analysis. Trizol reagent (Thermos Fisher Scientific, USA) was used to extract total RNA from the microtissues. Using the Revert Aid First Strand cDNA Synthesis Kit (Thermo Fisher, Massachusetts, USA) and KAPA SYBR FAST qPCR Kit Master Mix (KAPA Biosystems, USA), the expression of four genes, including OCN, Runx2, BMP-2 and Smad1 was assessed. R-β-actin was used as the internal control for mRNAs expression. All primer sequences used are shown in **Table S2**. Amplification conditions were as previously described 30.

### Ectopic bone formation in rat subcutaneous model

For ectopic bone formation experiment, 6 male Sprague Dawley rats were used. The experimental time is about 25 min for each rat. Six samples were implanted per rat.

After 7 days of expansion and 21 days of osteo-differentiation in the mini-capsule bioreactor, the microtissues-based TEBGs were collected from the honeycomb like chamber and transferred to PDMS chamber for subcutaneous implantation. Equal number (30) of core-shell/gelatin micro-scaffolds were put into PDMS chambers as the control. Six male Sprague Dawley rats aged 4 weeks were anesthetized with 90 mg/kg of Ketamine and 9 mg/kg of Xylazine. Six subcutaneous pockets were made on the back of each rat, and TEBGs of different groups were implanted randomly into the pockets as described previously 31.

Four weeks after implantation, the implanted samples were harvested and fixed in paraformaldehyde for 24 h. All specimens were subjected to micro-CT analysis (µ-80, Scanco Medical, Zurich, Switzerland) as described previously 32. We set a cylinder with the same size as the PDMS chamber (diameter: 5 mm; height: 2 mm) as the ROI. VG studio (Volume Graphics GmbH, Heidelberg, Germany) was used to reconstruct the scanned data and establish 3D models (Mean threshold value=226). The bone volume (BV) and bone volume/ tissue volume (BV/TV) were evaluated using the micro-CT assistant software. The specimens were then decalcified in 10% Ethylene Diamine Tetra Acetic Acid (EDTA) for 2 weeks, then dehydrated by a series of ethanol concentrations. The specimens were then embedded in paraffin and cut into 5 μm sections, followed by Hematoxylin and Eosin staining (HE) and immunohistochemical staining for CD31 as previously described 32. Quantitative analysis of the CD31 positive staining was evaluated as described previously 33.

### In vivo orthotopic bone formation in rats

A cranial defect rat model was used in 32 male Sprague Dawley rats. The experimental time is about 35 min for a rat. Two samples were implanted per rat.

For cranial defect repair assessment, 32 male adult Sprague Dawley rats aged 4 weeks and weighing 200-250 g were divided into two groups (n = 8 rats per group). Two critical sized cranial defects (diameter: 5.0mm) were drilled into each rat below the narcotism. The left-side defects of group A were filled with core-shell microtissues-based TEBG, and the right-side defects were filled with gelatin microtissues-based TEBG. In group B, the left-side defects were filled with core-shell micro-scaffolds-based TEBG and the right-side defects were filled with gelatin micro-scaffolds-based TEBG.

### Micro-Computed Tomography

Implanted specimens were harvested after 4 weeks and 12 weeks. The micro-CT analysis was then performed to evaluate the bone volume in all groups. Analysis of BV, BV/TV and bone mineral density (BMD) was performed using CTAn software.

### Histology & immunohistochemical analysis of col-1 and OCN

**HE and Masson's Trichrome staining**: After the fixation and decalcification process described above, samples were dehydrated by a series of ethanol concentrations 33. The specimens were then embedded in paraffin and cut into 5 μm sections, stained with Hematoxylin and Eosin (Sigma, St. Louis, USA) for tissue morphology examination and Masson's Trichrome (Sigma, St. Louis, USA) for neo-ossification.

**Immunohistochemical staining of col-1 and OCN**: The ECM components of new bone tissue were evaluated by immunohistochemical staining of type 1 collagen (col-1) and osteocalcin (OCN) as described previously 34. Sections were blocked by 3% BSA for 30 min, then incubated with col-1 and OCN monoclonal antibodies (Abcam, Cambridge, UK). Finally, images were captured using a phase contrast microscopy (Ni-E, Nikon, Tokyo, Japan).

### Statistical analyses

All data are presented as mean ±SD and were analyzed using Student's t-tests and one-way analysis of variance (ANOVA) test followed by post hoc contrasts by Student-Newman- Keuls test. P-values less than 0.05 were considered statistically significant.

## Results

### Characterization of core-shell micro-scaffolds and gelatin micro-scaffolds

**Figure 2:**
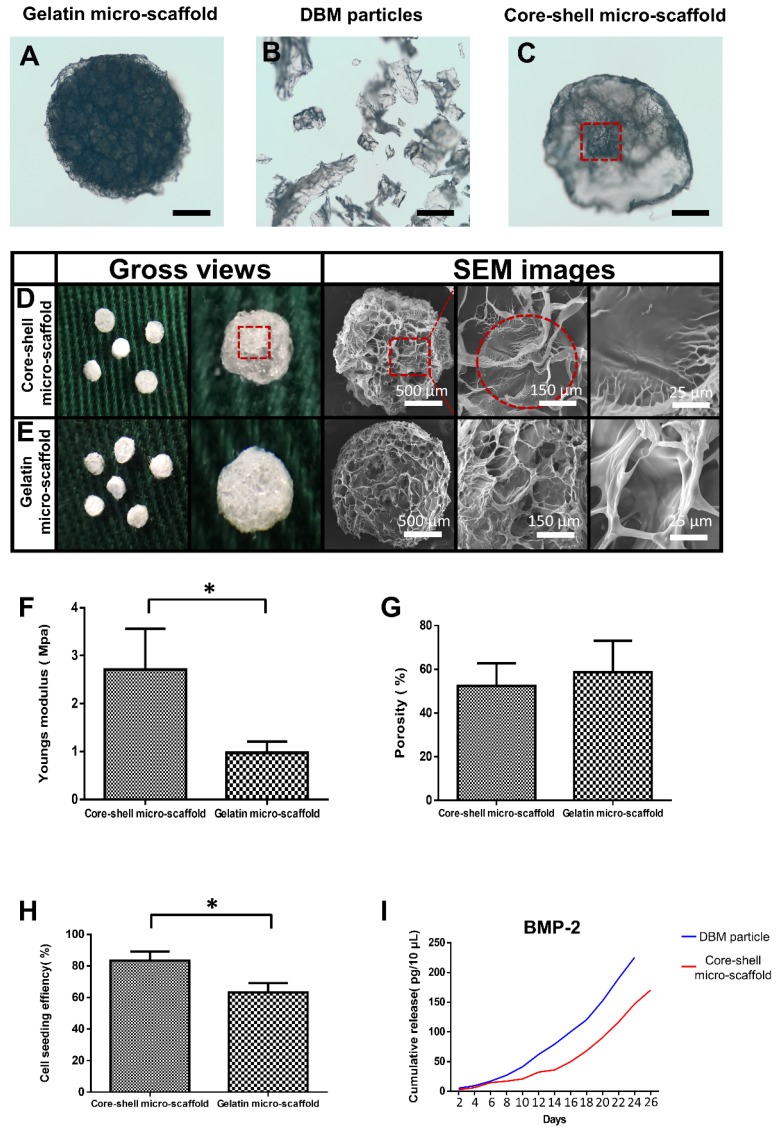
Characterization of core-shell micro-scaffolds and gelatin micro-scaffolds, respectively. (A-C) The outline of gelatin micro-scaffold, DBM particles and core-shell micro-scaffold were observed under a phase contrast microscope. (D, E) SEM images of core-shell micro-scaffolds and gelatin micro-scaffolds. (Red dotted circle marked the DBM core in core-shell micro-scaffolds) (F) Young's modulus of core-shell micro-scaffold was nearly triple that of gelatin micro-scaffold. (G) The porosities of core-shell micro-scaffold and gelatin micro-scaffold were of no significance. (H) Cell seeding efficiency of core-shell micro-scaffold and gelatin micro-scaffold. (I) Controlled release of BMP-2 on core-shell micro-scaffold and DBM particle during 26 days. (*: p < 0.05, scale bars in A, B, C: 500 μm)

The fabricated gelatin micro-scaffolds, DBM particles and core-shell micro-scaffolds were observed under a phase contrast microscope (**Figure 2A, Figure 2B, Figure 2C**). Gelatin micro-scaffold exhibited a uniformly distributed open porous structure, while DBM were irregular particles with lengths ranging 300-500 μm (**Figure S1**). The structure of core-shell micro-scaffold was composed of DBM particles positioned in the center as a core and enclosed by a gelatin shell. To further investigate the microstructures of the micro-scaffolds, SEM images were taken. Compared to gelatin micro-scaffolds, DBM particle was embedded in the center of core-shell micro-scaffolds and a layer of open porous gelatin shell was observed on the surface (**Figure 2D, Figure 2E**). The number of DBM particles in each core-shell micro-scaffold was counted, which indicated that most of them have only one DBM core (**Figure S2**). Due to the DBM core, core-shell micro-scaffolds showed a relatively higher Young's modulus than gelatin micro-scaffolds, while the porosity of both micro-scaffolds were of no significance (**Figure 2F, Figure 2G**). This means that core-shell micro-scaffold have better mechanical properties than gelatin micro-scaffold, which may be beneficial in application as TEBGs scaffold. The pore diameter of core-shell micro-scaffolds (about 70 μm) was smaller than that of gelatin micro-scaffolds (about 80 μm). (**Figure S3**) This may be caused by the DBM core in the middle squeezing the surrounded gelatin shell. The bioactivity of core-shell micro-scaffold and gelatin micro-scaffold were then compared by testing the cell seeding efficiency and cumulative release of BMP-2. BMSCs were pipetted and could be automatically adhered to core-shell/gelatin micro-scaffolds. The cell seeding efficiency of core-shell micro-scaffolds was between 80%-85%, better than that of gelatin micro-scaffolds, indicating that core-shell micro-scaffolds were more suitable for cell adhesion (**Figure 2H**). Cumulative release of BMP-2 in the PBS rinsing which signifies the quality of DBM particles/core-shell micro-scaffolds was determined during a 26-day period. Compared to DBM particles, core-shell micro-scaffolds released BMP-2 in a more sustained and controllable manner (**Figure 2I**).

### Cell proliferation in microtissues

**Figure 3:**
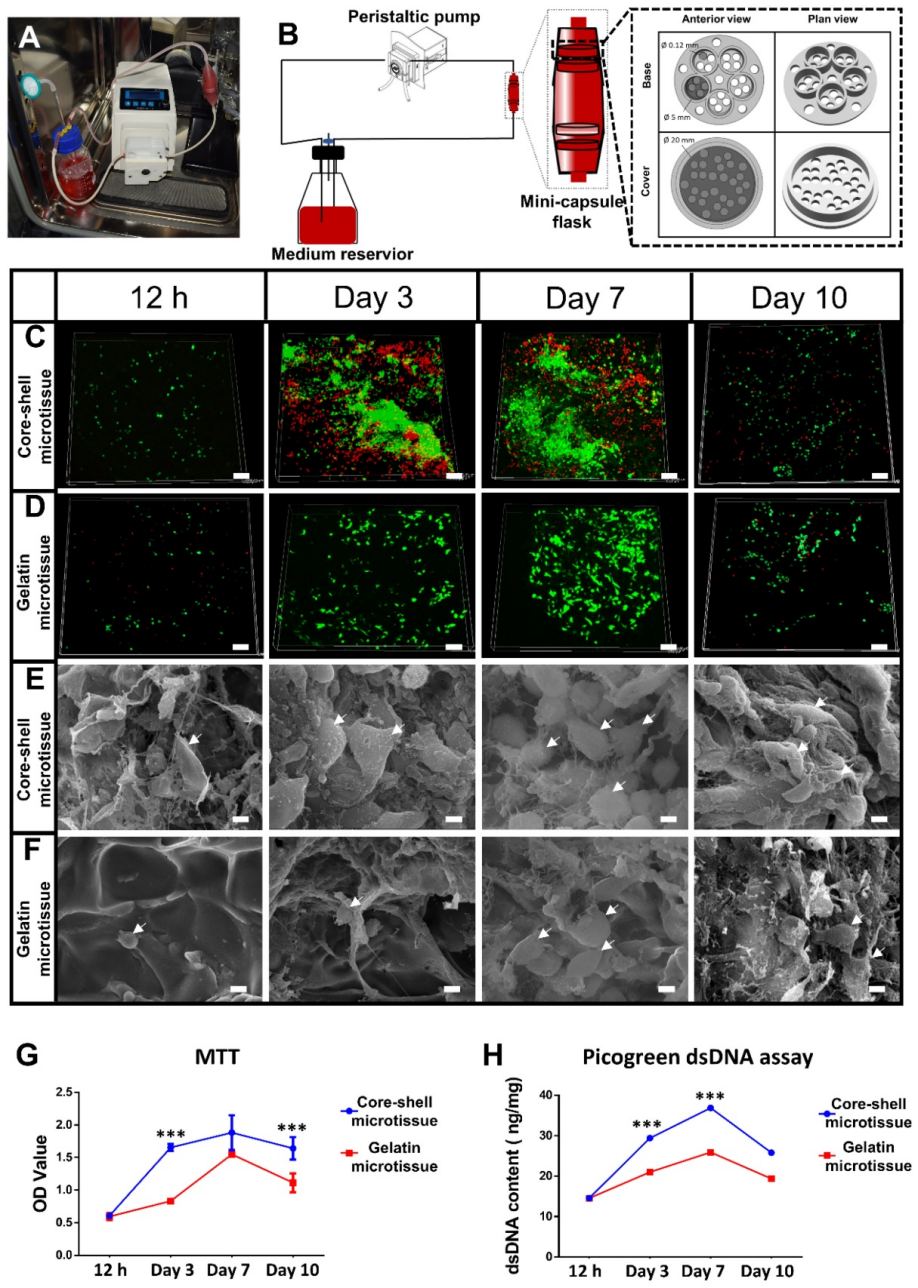
Dynamic perfusion culture of BMSCs expansion on core-shell microtissues and gelatin microtissues. (A, B) General view and schematic diagram of mini-capsule perfusion bioreactor. (C, D) FDA/PI staining of seeded BMSCs on core-shell microtissues and gelatin microtissues, wherein green fluorescence indicated cytoplasm of live cells and red fluorescence indicated nucleus of dead cells. (E, F) SEM images of BMSCs on core-shell microtissues and gelatin microtissues (white arrows indicate spread BMSCs). (G, H) MTT assay and dsDNA content of BMSCs on core-shell microtissues and gelatin microtissues during 10 days of dynamic expansion. (***: p < 0.001, scale bars in C, D: 150 μm, scale bars in E, F: 10 μm)

To achieve a large amount of BMSCs for better treatment efficacy, a mini-capsule bioreactor was designed and fabricated specifically for microtissues. The mini-capsule bioreactor was composed of three discrete parts as described. The gross view and design drawing of the mini-capsule bioreactor was shown in **Figure 3A** and **Figure 3B**. A honeycomb like chamber (Diameter: 5 mm, diameter of pores: 0.12 mm) was developed for holding microtissues, which would be assembled as a TEBG. The viability of seeded BMSCs was evaluated using FDA/PI assay. More live cells were found in core-shell microtissues during 10 days perfusion culture (**Figure 3C, Figure 3D, Figure S4**). SEM images showed that BMSCs on core-shell microtissues were fully spread and secreted more extracellular matrix than those on gelatin microtissues (**Figure 3E, Figure 3F**). Quantification analysis of cell activity on microtissues was determined by MTT assay, wherein BMSCs on core-shell microtissues showed a higher OD value (**Figure 3G**). Quantification of dsDNA content was performed to assess the expansion efficiency of BMSCs on microtissues during the perfusion culture. Consistent with MTT assay, the dsDNA content of BMSCs on core-shell microtissues was higher than that of gelatin microtissues (**Figure 3H**). The curves of MTT and dsDNA content of core-shell microtissue reached the peak at day 7, thus, osteogenic medium was added at day 7 when preparing for the in vivo experiments.

### Osteo-differentiation of BMSCs on microtissues

**Figure 4:**
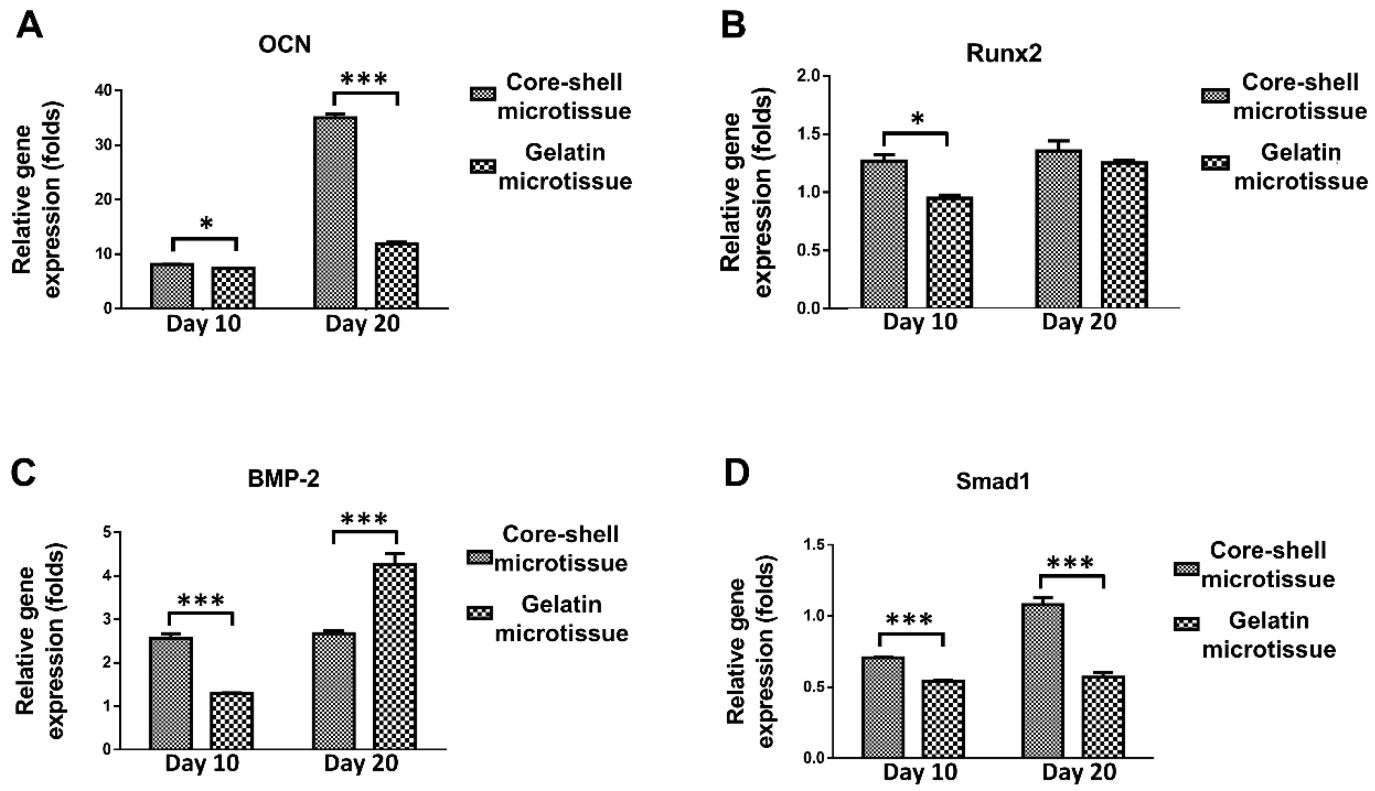
The relative expression of bone related genes after changing osteogenic medium at different time points by qRT-PCR. Samples were normalized by R-β-actin. (A, D) BMSCs on core-shell microtissues showed higher gene expressions of OCN and Smad1 compared to gelatin microtissues at day 20. (B, C) BMSCs on core-shell microtissues showed higher gene expressions of Runx2 and BMP-2 than gelatin microtissues at day 10. (*: p < 0.05, ***: p < 0.001)

**Figure 5:**
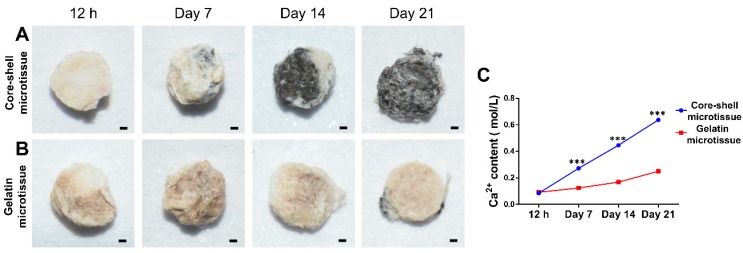
Comparison of osteogenic potential of core-shell microtissues and gelatin microtissues. (A, B) Von kossa assay of core-shell microtissues and gelatin microtissues during 21 days of osteo-differentiation period. (Black area indicates the mineralized modules) (C) Quantification of Ca^2+^ content of core-shell microtissues and gelatin microtissues during 21 days of osteo-differentiation period. (***: p < 0.001, scale bars in A, B: 150 μm)

The relative expression of bone related genes including OCN, Rux2, BMP-2 and Smad1 were evaluated at day 10 and day 20 (**Figure 4**). BMSCs on core-shell microtissues showed higher gene expressions of OCN and Smad1 compared to gelatin microtissues at day 20 (**Figure 4A, Figure 4D**). BMSCs on core-shell microtissues showed higher gene expressions of Runx2 and BMP-2 than gelatin microtissues at day 10 (**Figure 4B, Figure 4C**) After 7 days of expansion, the medium in the perfusion bioreactor was replaced with osteo-differentiation medium and cultured for 21 days. After 21 days of osteo-differentiation culture, von Kossa assay demonstrated more mineralized modules on core-shell microtissues than gelatin microtissues (**Figure 5A, Figure 5B**). Further, quantification of calcium content revealed that mineralized tissues on core-shell microtissues kept raising in 21 days and more calcium was deposited than gelatin microtissues (**Figure 5C**).

### Ectopic bone formation of core-shell/gelatin microtissues based TEBGs

**Figure 6:**
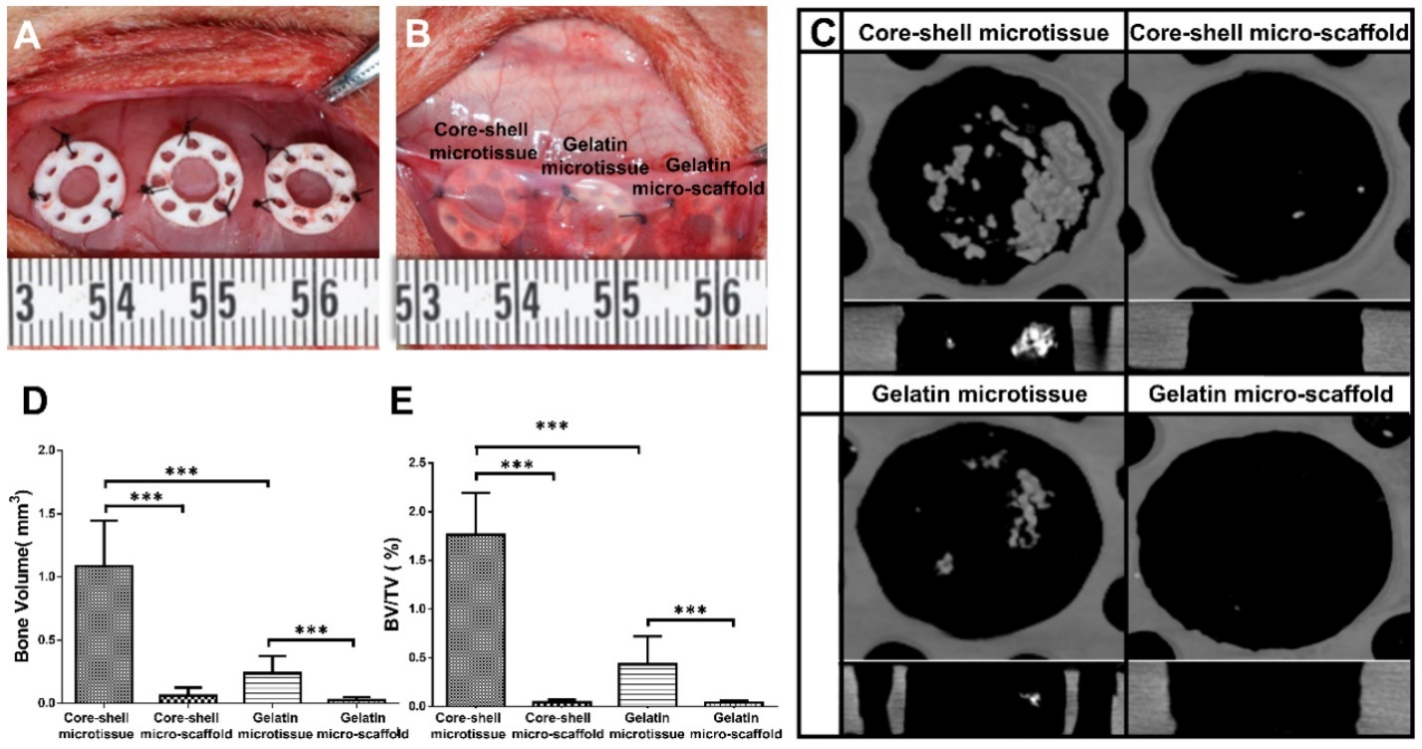
Ectopic osteogenesis of core-shell microtissues. (A, B) TEBGs of different groups were put inside PDMS chambers fixed on the back of rat, then covered with subcutaneous tissue. (C) Reconstructed micro-CT images of specimens after 4 weeks implantation. (D, E) Quantitative analyses of the BV and BV/TV ratio of different group of explants after 4 weeks implantation. (***: p < 0.001).

**Figure 7:**
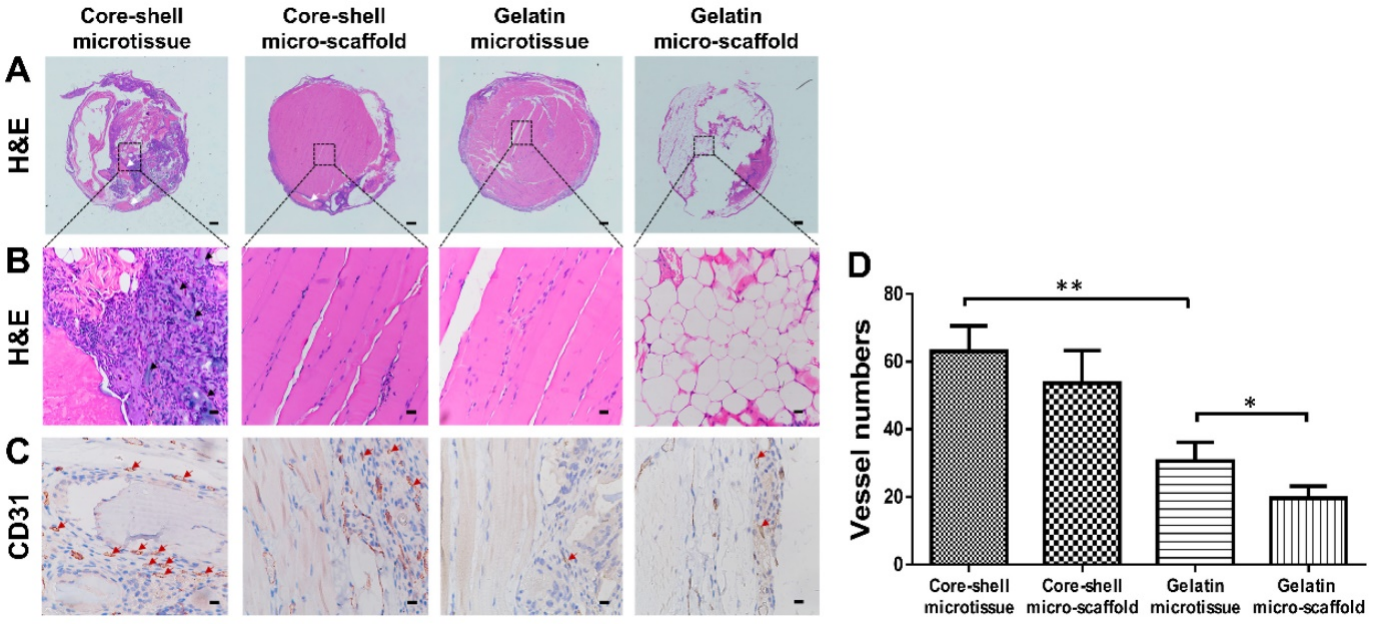
H&E and immunohistochemical staining of CD31 in the subcutaneous model of different groups after 4 weeks implantation. (A, B) H&E staining found that core-shell microtissue has the most amount of new bone tissue compared to other groups (White arrows indicate remained DBM particles, black arrows indicate new bone tissue). (C) Immunohistostaining of CD31 showed that core-shell microtissues formed the most amount of newly capillaries (Red arrows indicate newly formed vessels). (D) The mean vessel numbers quantified using Image-Pro Plus 6.0 software. (*: p < 0.05, **: p < 0.01, scale bars in A: 500 μm, scale bars in B: 100 μm, scale bars in C: 25 μm)

After expansion and osteo-differentiation, core-shell/gelatin microtissues based TEBGs were implanted subcutaneously in rat to detect the ectopic bone formation capacity (**Figure 6A, Figure 6B**). Core-shell/gelatin micro-scaffolds-based TEBGs were also implanted as the control experiment. Four weeks later, micro-CT images revealed that core-shell microtissues based TEBGs had the highest amount of calcification tissue (**Figure 6C**). BV and BV/TV of core-shell microtissues were nearly four-fold than gelatin microtissues (**Figure 6D, Figure 6E**). H&E staining of explants was performed to further confirm the micro-CT findings (**Figure 7A, Figure 7B**). At week 4, the highest amount of new bone tissues was formed in core-shell microtissues, in which the regenerated bone tissue distributed evenly throughout the whole explant. Immunohistochemical staining of CD 31 revealed that core-shell microtissues formed more capillaries than gelatin microtissues (**Figure 7C, Figure 7D**).

### Treatment of rat critical sized bone defect

**Figure 8:**
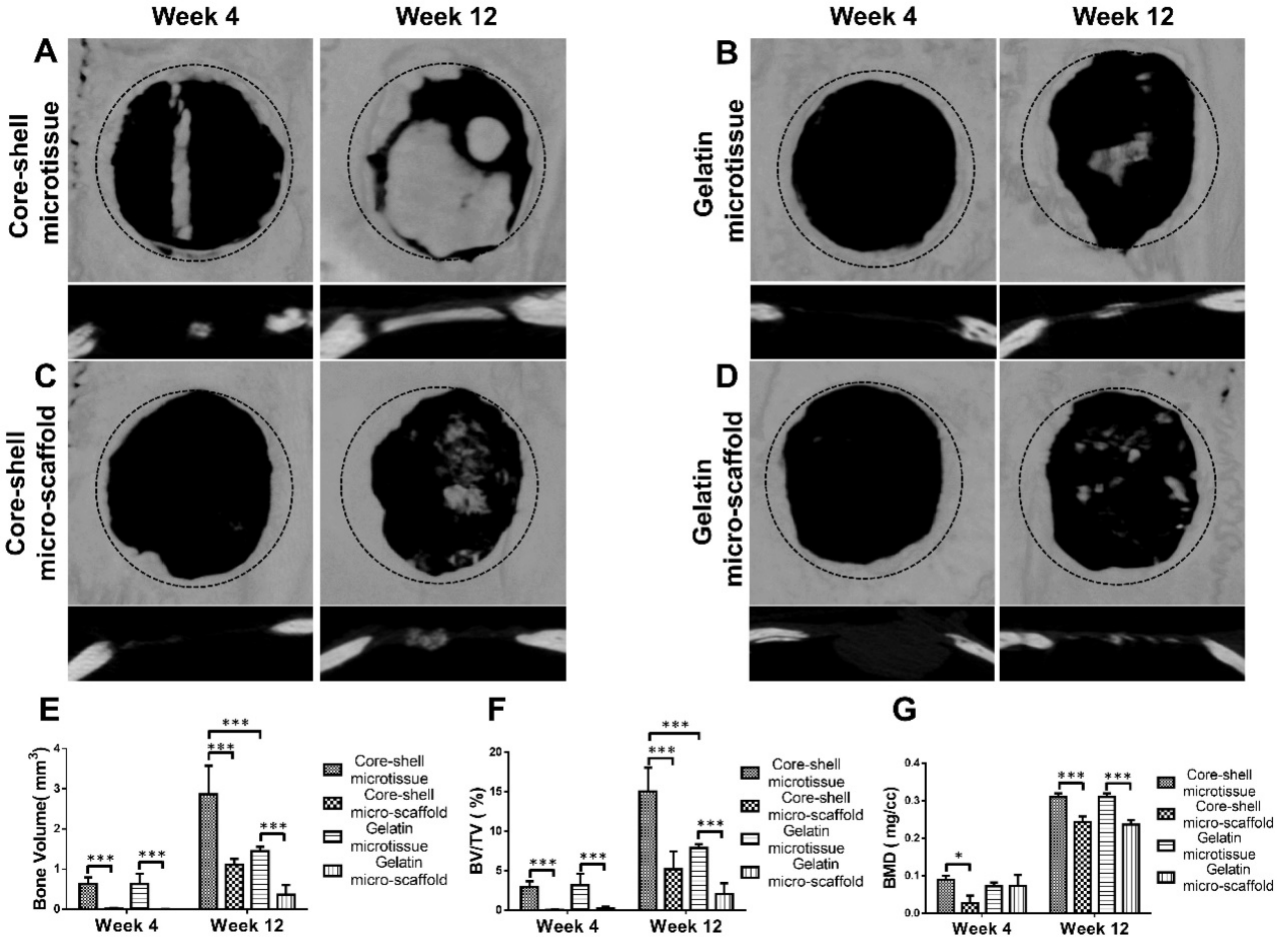
Micro-CT analysis of TEBGs for rat critical sized bone defect treatment. (A-D) 3D images of defect sites treated by core-shell microtissue/core-shell micro-scaffold/gelatin microtissue/gelatin micro-scaffold based TEBGs were reconstructed at 4 weeks and 12 weeks post treatment, respectively. (The black dotted circles indicate 5 mm critical sized bone defect). (E-G) Quantitative analyses of BV, BV/TV ratio and BMD at week 4 and week 12. (*: p < 0.05, ***: p < 0.001)

**Figure 9:**
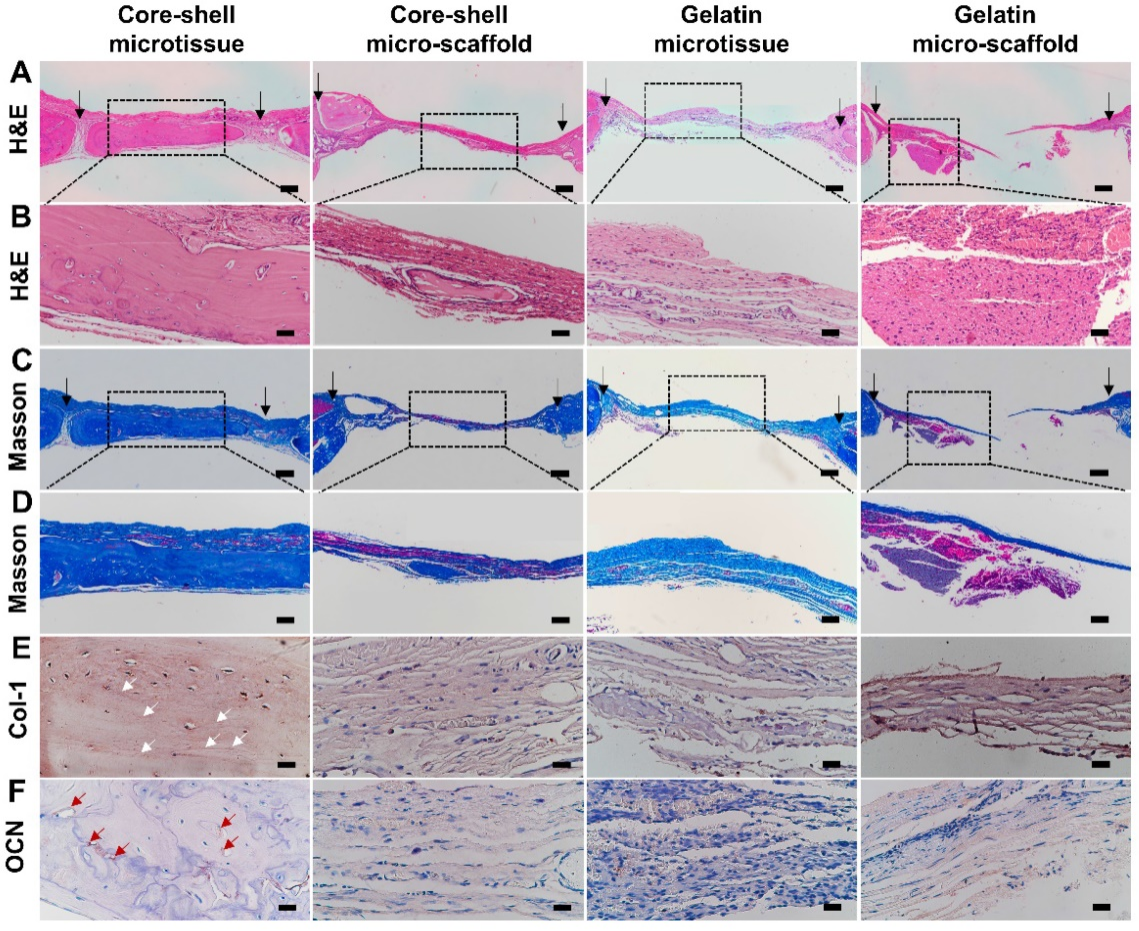
H&E, Masson's Trichrome staining and immuno-histochemical staining of col-1 and OCN of the defect sites after 12 weeks implantation. (A-D) Core-shell microtissue explant had a dense new bone tissue and the most calcium nodules in the defect site (Black arrows indicate the boarders of defect sites). (E, F) Immunohistochemical staining of col-1 and OCN (White arrows indicate the stained type 1 collagen, red arrows indicate OCN-positive cells). (Scale bars in A, C: 500 μm, scale bars in B: 100 μm, scale bars in D: 200 μm, scale bars in E, F: 50 μm)

Reconstructed micro-CT images after 12 weeks implantation found that core-shell microtissues formed the most amount of calcification tissue, while the defects of other three groups were partially filled with mineralized tissue (**Figure 8A, Figure 8B, Figure 8C, Figure 8D**). BV and BV/TV of core-shell microtissues were nearly 2-fold of the gelatin microtissues, with no significance of BMD between the two groups (**Figure 8E, Figure 8F, Figure 8G**). Histology of H&E and Masson's Trichrome staining of different group sections in cranial defects indicated that core-shell microtissues had the most amount of new bone tissue (**Figure 9A, Figure 9B, Figure 9C, Figure 9D**). And DBM was still remain in core-shell microtissues, which may be helpful for the integration of new bone in defect area. Immunohistochemical analysis of col-1 was performed to stain the linear type 1 collagen red, the results indicated that the ECM of core-shell microtissues in the newly formed bone tissue had more type 1 collagen than other groups (**Figure 9E**). Further, immunohistochemical staining of OCN (secreted by mature osteoblasts) revealed that more OCN-positive cells were found in core-shell microtissues (**Figure 9F**). These results showed that core-shell microtissues demonstrated better osteogenic activity than other groups in the rat cranial defect model.

## Discussion

Although reported microtissues can be applied in the regeneration of skin 35, as well as cartilage 36, their limited mechanical properties and low osteogenic activity make it unsuitable for bone regeneration. To achieve a better efficacy for bone regeneration, microtissues should be endowed with sufficient rigidity, enough shelter for cells during implantation, good osteo-inductivity and osteo-conductivity.

To improve the mechanical property and osteogenic activity of microtissues, DBM particle loaded with BMP-2 was introduced as the core material, with the advantages of following points: (1) The Young's modulus of core-shell micro-scaffolds was nearly triple that of gelatin micro-scaffolds, while their porosity was of no significance (**Figure 2F, Figure 2G**). The prior mechanical behavior of core-shell micro-scaffolds over gelatin micro-scaffolds might be owed to the supporting DBM core, which enhances the Young's modulus and ensures the mechanical stability of core-shell micro-scaffolds 37.

(2) BMP-2 was released from the core, and gradually reached the shell and exerted influences on BMSCs. The inner to outer release model guaranteed uniform and sustained effects on the BMSCs throughout the shell. Core-shell micro-scaffolds kept constant release of BMP-2 in 26 days, and the release trend was consistent with DBM particles (**Figure 2I**).

The controllable and sustained release of BMP-2 is more suitable for bone regeneration 38. Furthermore, BMP-2 release might be controlled by changing the network density or thickness of the shell 16. This is what we intend to explore in the following study. (3) Core-shell microtissues have a higher number of live cells with much more spread morphology and ECM accumulation than gelatin microtissues in the expansion stage (**Figure 3C, Figure 3D, Figure 3E, Figure 3F**). Picogreen dsDNA assay also revealed that the dsDNA content of core-shell microtissues was significantly higher than that of gelatin microtissues** (Figure 3H).** The better viability and ECM accumulation of BMSCs on core-shell microtissues might be caused by the stiff substrate provided by DBM core, which is helpful for cell spreading and proliferation through the modulation of mechanical information 39. Furthermore, substrates with higher Young's modulus can enhance cell proliferation through mechano-transduction of the mechanical information sensed from ECM 40.

The open porous structure of gelatin shell enabled the migration of seeded BMSCs from the surface to the inner part of the micro-scaffold. Once inside the micro-scaffold, the gelatin shell would act as a shelter to protect BMSCs from the mechanical insults and inflammatory factors through implantation. Yanan et al. found that the gelatin micro-cryogel could achieve the same treatment efficacy of critical limb ischemia by using 10 times less hMSCs dosage than simple injection of hMSCs 20. Daixu et al. made a highly open porous PHA microsphere as an injectable cell carrier, and demonstrated that it can act as a micro-Noah's Ark for cells due to the protection against stresses and changing microenvironment during injection 41. Micro-CT images and histology of H&E and Masson's Trichrome staining of core-shell microtissue group showed that the new bony tissue was formed mostly in the center of bone defect area, but no bony connection with bilateral cranial bones (**Figure 8, Figure 9**). These results suggest that the new bony tissue may be formed due to the survival of seeded BMSCs, followed by osteo-differentiation and secretion of mineralized matrix after implantation.

The results in this study showed that the core-shell microtissues had priorities over gelatin microtissues in terms of the osteogenic activity both in vitro and in vivo (**Figure 4-Figure 9**). The better osteogenic potential of core-shell microtissues might be due to the following reasons: (1) The BMPs are attached to the TGF-β superfamily. BMP-2 is regarded as one of the most significant molecules in skeletal regeneration with powerful osteo-inductivity, which is a strong osteo-inductive factor that accelerates osteo-differentiation of BMSCs by stimulating downstream Smad1/5/8 pathways 42. Here, the expression of Smad1 has been upregulated in core-shell microtissues (**Figure 4D**). It was speculated in this work that the downregulation of BMP-2 in core-shell microtissues might be attributed to the reason that BMP-2 represent early stage of osteo-differentiation (**Figure 4C**). Furthermore, BMP-2 is also a key factor in orchestrating the signaling pathway that regulates fracture repair in vivo 42. So, the higher amount of new bone tissue in core-shell microtissues might be owing to the BMP-2 loaded on DBM core. (2) The mechanical property of core-shell microtissues was similar to the native bone tissue, thus promoted the osteo-differentiation of BMSCs in vitro and accelerate surrounded MSCs attach, spread and differentiate into osteoblasts in vivo 43. Rigid materials can induce the osteo-differentiation of MCSs by upregulating the expression of Runx2 44. Since Runx2 is an early osteoblast marker 44, it was thus inferred that the higher expression of Runx2 in core-shell microtissues at day 10 might be due to the better mechanical property provided by DBM core (**Figure 4B**). When it comes to the later stage of osteo-differentiation period (day 20), the expression of Runx2 in different groups were of no significance. Here, we used rat cranial bone defect model to investigate the bone repair ability.

Sufficient number of seeded cells during implantation is needed to ensure healing of large bone defects 45. Traditional static culture in petri dish does not allow sufficient transportation of both nutrients and waste products (generally 200-250 µm) 46. One way of solving the problem is the application of bioreactors. Bioreactors accelerate the flow perfusion of scaffolds, which enable an adequate exchange of nutrients and oxygen 47. In this study, a specialized mini-capsule perfusion bioreactor was developed for the construction of microtissues based TEBGs. The replicated perfusion chambers allowed high-throughput fabrication of TEBGs with the same size and shape of bone defects. Furthermore, microtissues in the same perfusion chamber were tightly packed, which promoted the integrated fabrication of TEBGs by improving the interactions between seeded BMSCs (**Figure 3B**). Overall, this mini-capsule bioreactor achieved the integrated fabrication of TEBG by dynamic expansion and osteo-differentiation, which reduced the procedures of repeated trypsinization and medium exchange to improve the fabrication efficiency and decrease the risk of contamination.

## Conclusion

In conclusion, a novel micro-stencil array chip method was developed to construct DBM/gelatin core-shell microtissue. In this way, DBM/gelatin microtissues revealed enhanced mechanical properties, biocompatibility and better osteogenic activity than simple gelatin microtissues. As far as we know, this is the first time to apply core-shell structure into microtissue. This core-shell microtissue may provide a new way for the construction of tissue engineered bone graft.

## Supplementary Material

Supplementary figures and tables.

Supplementary video.

## Abbreviations

TEBGs: tissue engineered bone grafts
3D: three-dimensional
BU: bottom-up
ECM: extracellular matrix
RGD: Arg-Gly-Asp
DBM: demineralized bone matrix
BMP-2: bone morphogenetic protein 2
PEG: polyethylene glycol
PCL: poly(caprolactone)
PMMA: polymethyl methacrylate
EDC: 1-(3-Dimethylaminopropyl)-3-ethylcarbodiimide hydrochloride
NHS: 0.4% N-Hydroxysuccinimide;1-hydroxypyrrolidine-2,5-dione
PDMS: polydimethylsiloxane
SEM: scanning electron microscopy
DMEM: Dulbecco's Modified Eagle's Medium
FBS: fetal bovine serum
PLA: polylactic acid
FDA: fluorescein diacetate
PI: propidium iodide
MTT: 3-(4,5-dimethylthiazolyl-2)-2,5-diphenyltetrazolium bromide
DMSO: dimethyl sulfoxide
EDTA: Ethylene Diamine Tetra acetic Acid
qRT-PCR: quantitative real-time polymerization chain reaction
Runx2: runt-related transcription factor 2
BMP-2: bone morphogenetic protein 2
ROI: regions of interest
BV: bone volume
TV: total volume
BMD: bone mineral density
SD: standard deviation
VEGF: vascular endothelial growth factor
FN: fibronectin
hMSCs: human adipose-derived mesenchymal stem cells
PHA: polyhydroxyalkanoate
TGF-β: transforming growth factor-β
AgNO3: silver nitrate
