# Supplementary material for: Biomimetic open porous structured core-shell microtissue with enhanced mechanical properties for bottom-up bone tissue engineering
Source: Theranostics. 2019 Jul 9;9(16):4663–77. doi: 10.7150/thno.34464 (PMC6643438; doi:10.7150/thno.34464)
Supplement: Supplementary file 1 — Supplementary figures and tables. [file thnov09p4663s1.pdf]

Supplementary data

Biomimetic open porous structured core-shell microtissue with superior mechanical properties for bottom-up bone tissue engineering

Chao Luo[a,#], Huimin Fang[a,#], Muran Zhou[a], Jialun Li[a], Xinyue Zhang[a], Shaokai Liu[a], Chuchao Zhou[a], Jinfei Hou[a], Huan He[a], Jiaming Sun[a,*], Zhenxing Wang[a,*]

a Department of Plastic Surgery, Union Hospital, Tongji Medical College, Huazhong University of Science and Technology, Wuhan 430022, China.

# These authors contributed equally to this work.

* Corresponding authors

Corresponding authors' e-mail addresses:

*Zhenxing Wang: wangzhenxing@hust.edu.cn

*Jiaming Sun: sunjiaming@hust.edu.cn

Table S1. Explanation of samples tested in the experiment.

Sample	Explanation
Core-shell micro-scaffold	Microgel fabricated by DBM-gelatin mixture
Core-shell microtissue	Cell-material composite of seeded BMSCs and core-shell micro-scaffold
Gelatin micro-scaffold	Microgel fabricated by simple gelatin solution
Gelatin microtissue	Cell-material composite of seeded BMSCs and gelatin micro-scaffold

Table S2. Primers sequences used in qRT-PCR.

Gene	Primer sequence
R-βactin-F	5'- TGCTATGTTGCCCTAGACTTCG-3'
R-βactin-R	3'- GTTGGCATAGAGGTCTTTACGG-5'
R-OCN-F	5'- CATCTATGGCACCACCGTTTA-3'
F-OCN-R	3'- CATCTATGGCACCACCGTTTA-5'
R-Runx2-F	5'- CAGTATGAGAGTAGGTGTCCCGC-3'
F-Runx2-R	3'- AAGAGGGGTAAGACTGGTCATAGG-5'
R-BMP-2-F	5'- AACACCGTGCTCAGCTTCCAT-3'
F-BMP-2-R	3'- GAAGAAGAAGCGTCGGGAAGT-5'
R-Smad1-F	5'- GACTGCCTCATGTCATTTATTGC-3'
F-Smad1-R	3'- CGCTTATAGTGGTAGGGATTGATG-5'

F: Forward; R: Reverse.

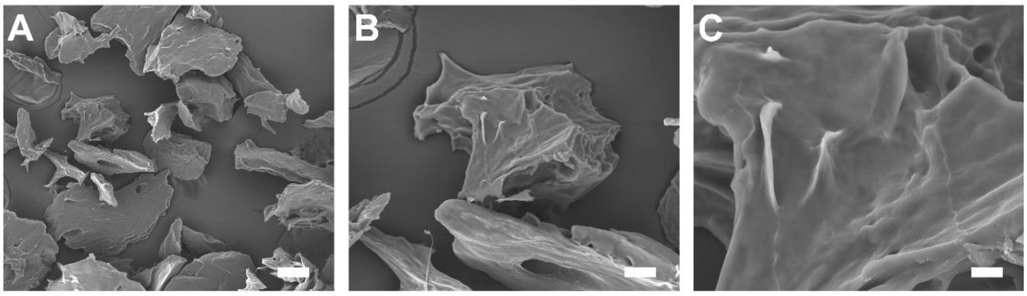

Figure S1 SEM images of DBM particles. (Scale bars in A: 125 μm, scale bars in B: 50 μm, scale bars in C: 12.5 μm)

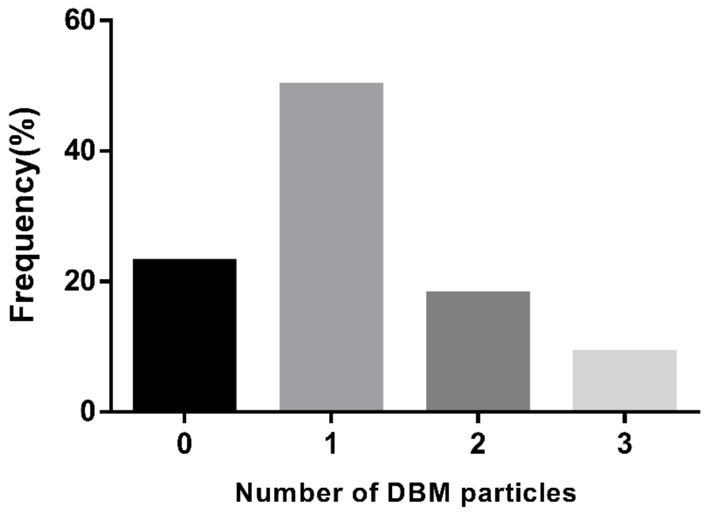

Figure S2 Number of DBM particles in each core-shell micro-scaffolds was counted. Approximately 50% core-shell micro-scaffolds have only one DBM particle as the core.

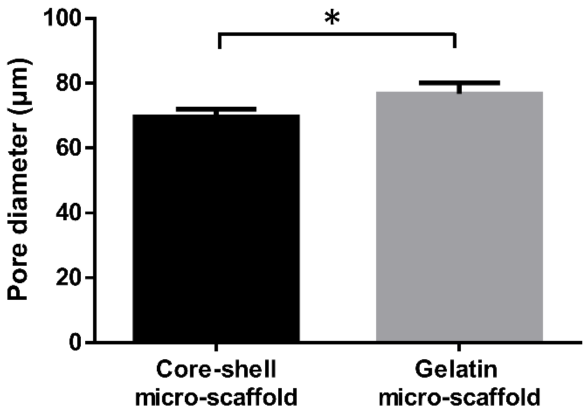

Figure S3 Pore diameter of core-shell micro-scaffold and gelatin micro-scaffold. (*: $p<0.05$)

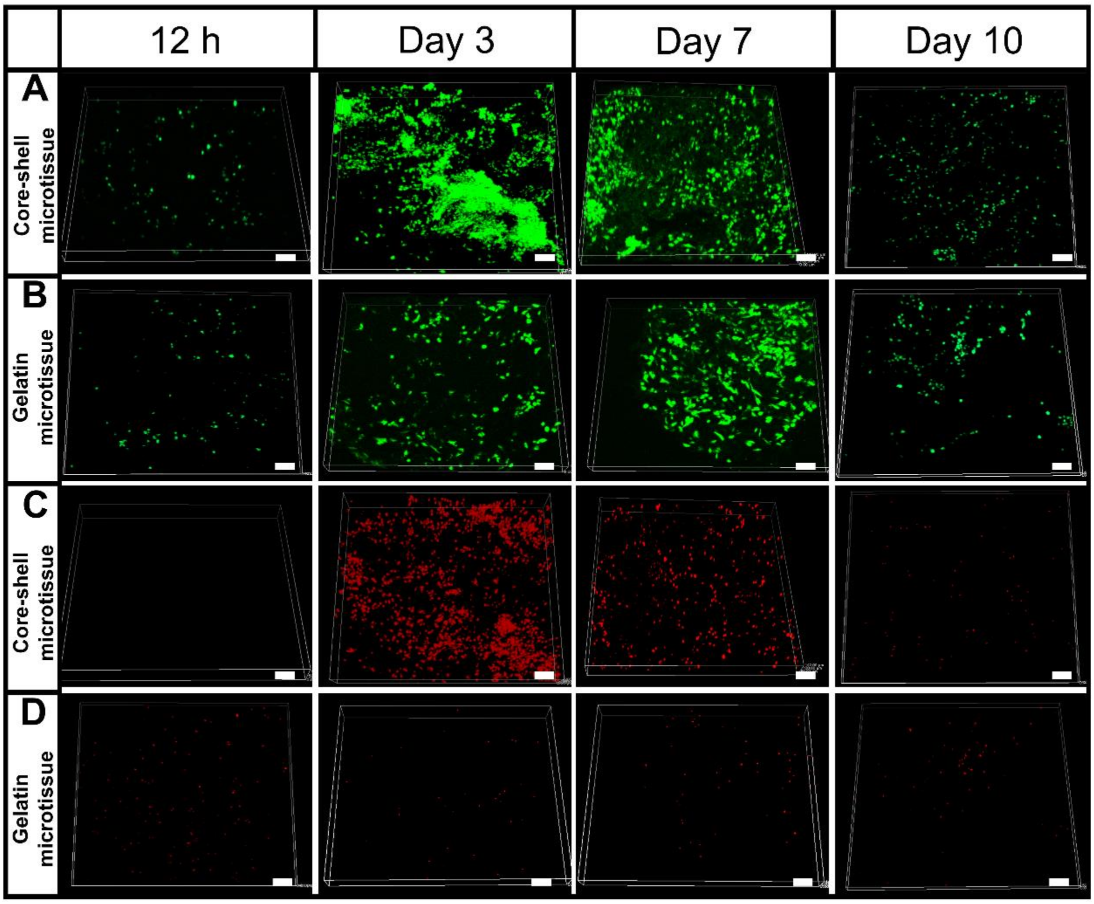

Figure S4 Confocal images of FDA staining and PI staining. (A, B) Confocal images of FDA staining of core-shell microtissue and gelatin microtissue, wherein green fluorescence indicated cytoplasm of live cells. (C, D) Confocal images of PI staining of core-shell microtissue and gelatin microtissue, wherein red fluorescence indicated nucleus of dead cells. (Scale bars: 150 μm)
